# Major Histocompatibility Complex Class I Haplotype Diversity in Chinese Rhesus Macaques

**DOI:** 10.1534/g3.113.006254

**Published:** 2013-07-01

**Authors:** Julie A. Karl, Patrick S. Bohn, Roger W. Wiseman, Francesca A. Nimityongskul, Simon M. Lank, Gabriel J. Starrett, David H. O’Connor

**Affiliations:** * Wisconsin National Primate Research Center, University of Wisconsin-Madison, Madison, Wisconsin 53715; † Department of Pathology and Laboratory Medicine, University of Wisconsin-Madison, Madison, Wisconsin 53705

**Keywords:** *Macaca mulatta*, MHC class I, RNA transcript-based haplotypes, immunogenetics

## Abstract

The use of Chinese-origin rhesus macaques (*Macaca mulatta*) for infectious disease immunity research is increasing despite the relative lack of major histocompatibility complex (MHC) class I immunogenetics information available for this population. We determined transcript-based MHC class I haplotypes for 385 Chinese rhesus macaques from five different experimental cohorts, providing a concise representation of the full complement of MHC class I major alleles expressed by each animal. In total, 123 *Mamu-A* and *Mamu-B* haplotypes were defined in the full Chinese rhesus macaque cohort. We then performed an analysis of haplotype frequencies across the experimental cohorts of Chinese rhesus macaques, as well as a comparison against a group of 96 Indian rhesus macaques. Notably, 35 of the 51 *Mamu-A* and *Mamu-B* haplotypes observed in Indian rhesus macaques were also detected in the Chinese population, with 85% of the 385 Chinese-origin rhesus macaques expressing at least one of these class I haplotypes. This unexpected conservation of Indian rhesus macaque MHC class I haplotypes in the Chinese rhesus macaque population suggests that immunologic insights originally gleaned from studies using Indian rhesus macaques may be more applicable to Chinese rhesus macaques than previously appreciated and may provide an opportunity for studies of CD8^+^ T-cell responses between populations. It may also be possible to extend these studies across multiple species of macaques, as we found evidence of shared ancestral haplotypes between Chinese rhesus and Mauritian cynomolgus macaques.

Macaque monkeys are used extensively to model and understand human diseases. Historically, rhesus macaques (*Macaca mulatta*; *Mamu*) of Indian origin have been preferred for such research. However, exportation of rhesus macaques from India was halted in 1978 (Southwick and Siddiqi 1994). As it became more difficult to obtain Indian rhesus macaques, researchers turned to other sources, including Chinese-origin rhesus macaques. Chinese rhesus macaques have been used to study infectious diseases including simian immunodeficiency virus/human immunodeficiency virus, avian influenza, malaria, plague, and severe acute respiratory syndrome, as well as studies of xenotransplantation, stem cell therapies, reproduction, and neurobiology (Shen *et al.* 2007; Chen *et al.* 2009, 2011, 2012; Choi *et al.* 2011; Liu *et al.* 2011; Tian *et al.* 2011; Wei *et al.* 2011; Hutnick *et al.* 2012; Li *et al.* 2012b; Ling *et al.* 2013; Mumbauer *et al.* 2013). Yet comparatively little is known about the immunogenetics of these animals. The genome of a single Chinese rhesus macaque was sequenced in 2011, but whole-genome sequencing technology struggles to accurately characterize highly polymorphic, duplicated regions such as the major histocompatibility complex (MHC) loci and killer immunoglobulin receptor loci (Fang *et al.* 2011).

The MHC is an exceptionally polymorphic region of the genome responsible for presentation of peptides to T cells (Bontrop 2006). Because of its pivotal role in immune response, understanding MHC genetics is essential for infectious disease research. Our group and others have shown that the repertoire of MHC class I alleles expressed by Chinese rhesus macaques is largely distinct from that observed in Indian rhesus macaques (Otting *et al.* 2007, 2008; Karl *et al.* 2008; Otting *et al.* ; Wu *et al.* 2008; Ma *et al.* 2009; Wiseman *et al.* 2009; Naruse *et al.* 2010; Doxiadis *et al.* 2011). Consequently, we inferred MHC class I−restricted CD8^+^ T-cell responses in these two populations would be unlikely to target many of the same epitopes.

Here we reconsider the relationship between MHC class I genetics of Chinese- and Indian-origin rhesus macaques by focusing on MHC class I transcript−defined haplotypes inherited together, rather than simply analyzing individual allelic variants. Most studies that currently control for the complex rhesus macaque MHC class I region only account for the presence of one or a few widely studied alleles (Mamu-A1*001, etc.), with no regard for the majority of alleles expressed by the animal. Analysis on a haplotype level as presented here provides a more global view of the MHC class I alleles transcribed by each animal, creating the potential for a greater degree of allele matching between animals within a study.

To define haplotypes with high resolution, we deep sequenced complementary DNA (cDNA) amplicons spanning most of exons 2−4 of *Mamu-A* and *Mamu-B* genes, the most polymorphic area of MHC class I corresponding to the peptide binding region. A total of 385 Chinese-origin rhesus macaques from five different experimental cohorts were examined, as well as 96 Indian-origin rhesus macaques from two distinct sources. Unexpectedly, we discovered that 35 of 51 of the *Mamu-A* and *Mamu-B* haplotypes observed in Indian rhesus macaques also were expressed by a significant majority of Chinese rhesus macaques. Conservation of these haplotypes suggests that functional CD8^+^ T-cell immunity to pathogens may be more similar between Chinese- and Indian-origin rhesus macaques than previously appreciated.

## Materials and Methods

### Animals

Whole blood and peripheral blood mononuclear cell samples from 385 Chinese rhesus macaques were obtained over the span of 5 yr from five different sources, including Battelle Biomedical Research Center, Etubics Corporation, the National Institute of Allergy and Infectious Diseases/National Institutes of Health, and Harlan Laboratories, Inc. Cohorts are detailed in Table 1. The core set of 51 Chinese rhesus macaque samples included 45 samples from cohort ChRh #1 and six samples from cohort ChRh #4. The expanded set of 334 Chinese rhesus macaque samples included an additional 142 animals from these two cohorts, as well as 192 animals from the three other sources. It is unknown from which provinces in China the animals or their ancestors were initially obtained, or any degree of relation between the animals sampled.

**Table 1:** Summary of Chinese- and Indian-origin rhesus macaque cohorts

Cohort ID	Total No. Animals	Total No. Chromosomes	Core Set No. Animals	Expanded Set No. Animals
ChRh #1	147	294	45	102*^a^*
ChRh #2	133	266	−	133
ChRh #3	48	96	−	48
ChRh #4	46	92	6	40*^a^*
ChRh #5	11	22	−	11
InRh #1	49	98	49	−
InRh #2	47	94	47	−

a Evaluated using the 248-bp primers; all other animals were amplified with the 638-bp primers.

Whole blood samples from 96 Indian rhesus macaques were obtained from the Wisconsin National Primate Research Center (WNPRC) and New England Primate Research Center (NEPRC) breeding colonies. The WNPRC cohort was selected to be unrelated based on pedigree records whereas the NEPRC cohort was taken from active adult breeding stock. Macaques at the WNPRC were cared for according to protocols approved by the University of Wisconsin-Madison Graduate School Animal Care and Use Committee, whereas macaques housed at the NEPRC were maintained in accordance with standards of the Association for Assessment and Accreditation of Laboratory Animal Care and the Harvard Medical School Animal Care and Use Committee.

### RNA isolation, cDNA synthesis, and polymerase chain reaction (PCR) amplification (normalization and pooling)

RNA was isolated from whole blood or peripheral blood mononuclear cell samples using the Roche MagNA Pure instrument and RNA High Performance kit (Roche, Indianapolis, IN), following manufacturer’s protocols (Supporting Information, File S1). Synthesis of cDNA and PCR amplification were performed as previously described, generating either a 248-bp or 638-bp genotyping amplicon (the shorter amplicon dates back to studies performed before Roche/454 sequencing technology advanced to ~500-bp read lengths) (Wiseman *et al.* 2009; Fernandez *et al.* 2011). The amplicons for each sample were tagged during primary PCR with a multiplex identifier sequence, a unique 10-bp tag for each sample (File S1). Amplification was confirmed by running PCR products on a Flash Gel (Lonza Group Ltd, Basel, Switzerland), and bead-based purification was performed using AMPure XP SPRI beads (Agencourt Bioscience Corporation, Beverly, MA) to remove primer dimers. Quantification of purified products was performed using the Quant-iT dsDNA HS Assay kit and a Qubit fluorometer (Invitrogen, Carlsbad, CA). Products were then normalized to 0.3 ng/μL and pooled in equal volumes.

### Sequencing

Sequencing for all amplicons was performed using Roche/454 second-generation methods following manufacturer’s protocols for emulsion PCR and pyrosequencing (Roche, Indianapolis, IN). The core set of 51 Chinese rhesus macaques and 96 Indian rhesus macaques were sequenced in a total of five runs on a GS Junior pyrosequencing instrument. The expanded set of 334 Chinese rhesus macaques were sequenced on a combination of GS Junior and GS FLX platforms; both platforms utilize the same basic chemistry, differing only in scale of the run (a single GS Junior run is ~1/8 the scale of a full GS FLX run).

### Data analysis

For the core set of 51 Chinese-origin and 96 Indian-origin rhesus macaques, sequences were analyzed by trimming all sequences from the 3′ end to fixed 430-bp lengths, binning sequences into separate animal groups by parsing for the unique multiplex identifier tags, then creating uni-directional contigs of sequence reads assembled at 100% stringency using the program Geneious Pro (BioLegend, San Diego, CA). FASTA format sequences of all contigs for each sample were then exported, and bidirectional reads were assembled at 100% stringency using CodonCode Aligner (CodonCode, Dedham, MA). Bidirectional contigs were interrogated against a curated database of known rhesus macaque MHC class I alleles using the Basic Local Alignment Search Tool (*i.e.*, BLAST), and novel alleles were submitted to GenBank (KC205288-KC205396). Putative haplotypes were inferred by looking for combinations of shared alleles between animals (File S1). Analyses of the *Mamu-A* and *Mamu-B* regions were performed independently, since these gene clusters are separated by ~1.2 million base pairs and recombination is relatively common at the population level (Daza-Vamenta *et al.* 2004).

Once putative haplotypes were defined, a simpler workflow was used to assign haplotype designations to the additional 334 Chinese rhesus macaque samples from the expanded set. All individual sequence reads obtained for each sample were directly compared against our rhesus macaque MHC class I allele database using BLAST-like analysis tool (*i.e.*, BLAT) in a local instance of Galaxy (Penn State, State College, PA; Emory, Atlanta, GA), following the data analysis workflow detailed in Figure S1. Haplotypes were assigned by identifying groups of shared major alleles from the previously defined putative haplotypes.

## Results

### Haplotype definition based on *Mamu-A* and *Mamu-B* transcripts

**Figure 1:**
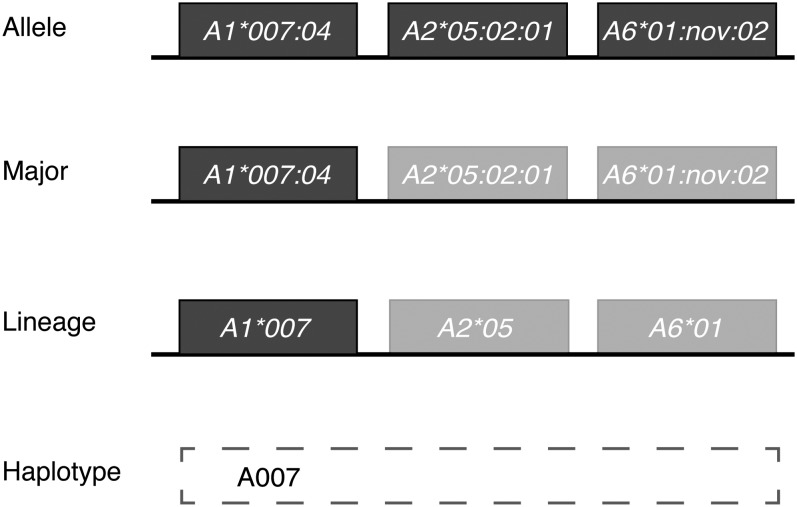
Simplification of MHC class I allele calls from full allele down to haplotype designation. Schematic representation of reducing the A007 haplotype observed in Chinese-origin rhesus macaques, from full allele name to major alleles to lineage designation to final haplotype call.

The unparalleled complexity of the nonhuman primate MHC class I region makes it difficult to describe simply the full diversity of class I alleles expressed by an animal. Macaques may encode as many as 30 distinct MHC class I transcripts expressed at widely differing steady state levels in peripheral blood. However, combinations of distinct alleles are inherited together on a chromosome as haplotypes, making it possible to assign a simplified name (*i.e.*, haplotype A007) to each specific combination of alleles. Here, unique haplotypes were defined on the basis of transcriptionally abundant “major” *Mamu-A* and *Mamu-B* alleles; an example of this simplification for the A007 haplotype is illustrated in Figure 1. Our operational criterion for defining a “major” allele is that it must exceed a 4% transcript abundance relative to all class I transcripts identified per animal. We previously showed that CD8^+^ T-cell responses are principally restricted by highly transcribed major MHC class I alleles expressed in macaques, so we believe functionally defining haplotypes based on configurations of major alleles is appropriate for assessing conservation of MHC alleles involved in antigen presentation to CD8^+^ T cells (Budde *et al.* 2011).

To further streamline haplotype definitions, only lineage level allele names were considered (commonly referred to as two-digit resolution in human HLA genotyping, *e.g.*, all Mamu-A1*007 allelic variants were grouped with the designation Mamu-A1*007; Figure 1). Alleles are named by the nonhuman primate Immuno Polymorphism Database-MHC group based on similarity to other known macaque MHC class I alleles, such that alleles sharing a common lineage level designation are identical or nearly so within the peptide binding regions.

A total of 123 unique haplotypic configurations were defined in the full cohort of 385 Chinese rhesus macaques, consisting of 53 distinct *Mamu-A* haplotypes and 70 *Mamu-B* haplotypes (Table S1). Names were assigned to each haplotype based on the presence of what we call a “diagnostic major” allele. *Mamu-A* region haplotypes typically contained a single major allele, usually from the *Mamu-A1* locus, so that allele was considered the “diagnostic major.” A few of the *Mamu-A* haplotypes and most of the *Mamu-B* haplotypes expressed two or more major alleles; for these haplotypes, the most transcriptionally abundant allele expressed on the haplotype was typically considered the “diagnostic major” allele. Occasionally the “diagnostic major” allele was selected based on known biological significance. For instance, even though the Mamu-B*017 allele was not typically the most abundant transcript, it was considered the “diagnostic major” due to its known association with spontaneous control of SIV (Yant *et al.* 2006). Different versions of a base haplotype, denoted with a lowercase letter following the haplotype name (for instance, B001a and B001b), were designated to indicate differences in the complement of major transcripts accompanying a shared diagnostic allele. By this method of haplotype definition, four simple haplotype names represent the full complement of MHC class I major alleles expressed by each macaque (Table S2).

### Frequencies of haplotypes across different Chinese rhesus macaque cohorts

**Figure 2:**
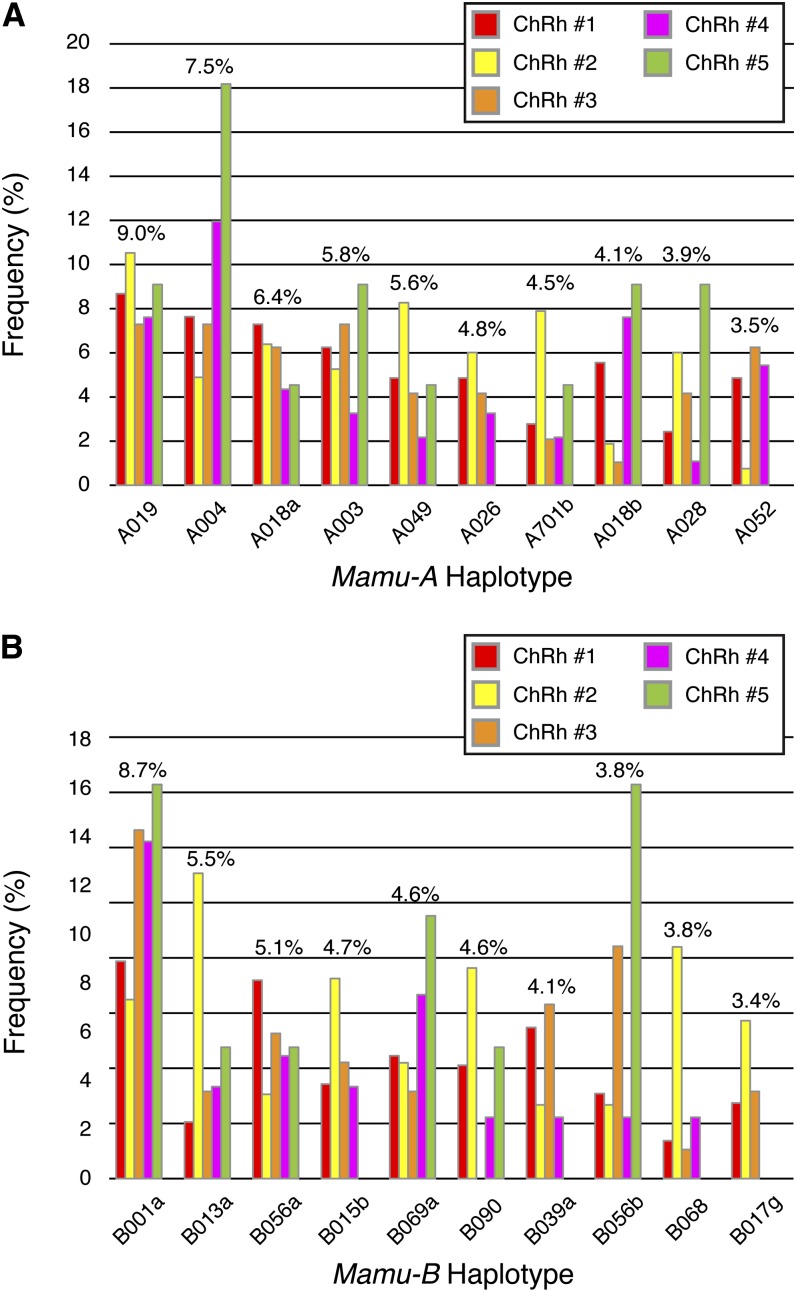
Frequency distribution by cohort for the 20 overall most frequent haplotypes in Chinese rhesus macaques. (A) Frequencies of the 10 overall most abundant Chinese rhesus macaque *Mamu-A* haplotypes, separated by cohort, within the full cohort of 385 Chinese rhesus macaques. Average frequency across Chinese rhesus macaque cohorts is listed above the columns. Frequencies were calculated based on total number of chromosomes examined (770 Chinese chromosomes) to account for each animal having two haplotypes for each region. (B) Frequencies of the 10 overall most abundant *Mamu-B* haplotypes, separated by cohort. Average frequency across all five Chinese rhesus macaque cohorts is listed above the columns.

Because the geographical source of macaques within China has been indicated as a factor in frequency of specific MHC class I alleles, we wanted to explore the differences in chromosomal haplotype frequencies between our five experimental cohorts (Li *et al.* 2012a; Liu *et al.* 2013). After identifying the 20 most common Chinese rhesus macaque *Mamu-A* and *Mamu-B* haplotypes across the data set as a whole, we examined the differences in the frequencies for those haplotypes between the five cohorts (Figure 2). Frequencies were calculated based on the total number of chromosomes examined (770 Chinese rhesus macaque chromosomes), to account for each animal expressing pairs of *Mamu-A* and *Mamu-B* haplotypes. Some haplotype frequencies, such as A019, A018a, and B056a, were relatively consistent across the cohorts; however, some frequencies varied greatly from cohort to cohort. One example is the A004 haplotype, which was observed in ~18% of the total chromosomes examined for cohort ChRh #5, but only ~5% for cohort ChRh #2. Overall, frequency differences between cohorts were more pronounced for the *Mamu-B* haplotypes, and some cohorts did not contain one or more of the 20 most common *Mamu-A* and *Mamu-B* haplotypes. This finding is not unexpected for cohort ChRh #5, because it only contained 11 macaques. The remaining cohorts each contained at least 46 macaques, minimizing the effect of sample size on observed frequencies in these cohorts. These results emphasize the point that not all research cohorts of Chinese rhesus macaques are equal from the standpoint of MHC class I diversity, as the complement of major MHC class I alleles expressed by Chinese rhesus macaques can vary greatly between different sample groups. However, despite frequency differences, common haplotypes can still be identified across all five cohorts.

### Haplotype comparison between Chinese and Indian rhesus macaques

**Figure 3:**
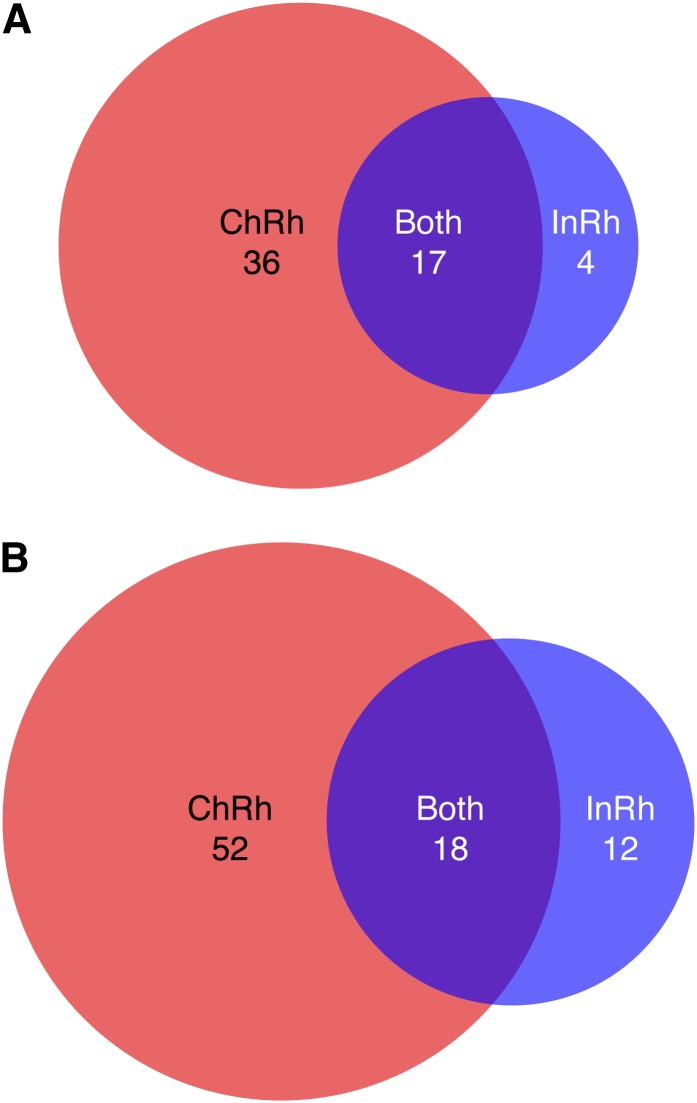
Haplotype distribution between Chinese and Indian rhesus macaques by region. (A) Distribution of *Mamu-A* haplotypes by population within the full cohort of 385 Chinese rhesus macaques and 96 Indian rhesus macaques. (B) Distribution of *Mamu-B* haplotypes by population within the full cohort.

We next performed MHC class I haplotype analysis on a group of 96 Indian rhesus macaques, allowing for haplotype comparison between the two geographically isolated populations. In total, 51 haplotypes were identified in the Indian rhesus macaque cohort, including 21 *Mamu-A* haplotypes and 30 *Mamu-B* haplotypes. Interestingly, only 16 haplotypes (four *Mamu-A* and 12 *Mamu-B* haplotypes) were unique to Indian rhesus macaques in this comparison (Figure 3). These findings indicate that although the allelic variation between Chinese- and Indian-origin rhesus macaques is largely distinct (Table S3), the two populations frequently inherit shared combinations of closely related allelic variants. Overall, 35 of the 51 MHC class I haplotypes present within Indian rhesus macaques have closely related homologs in the Chinese population.

**Figure 4:**
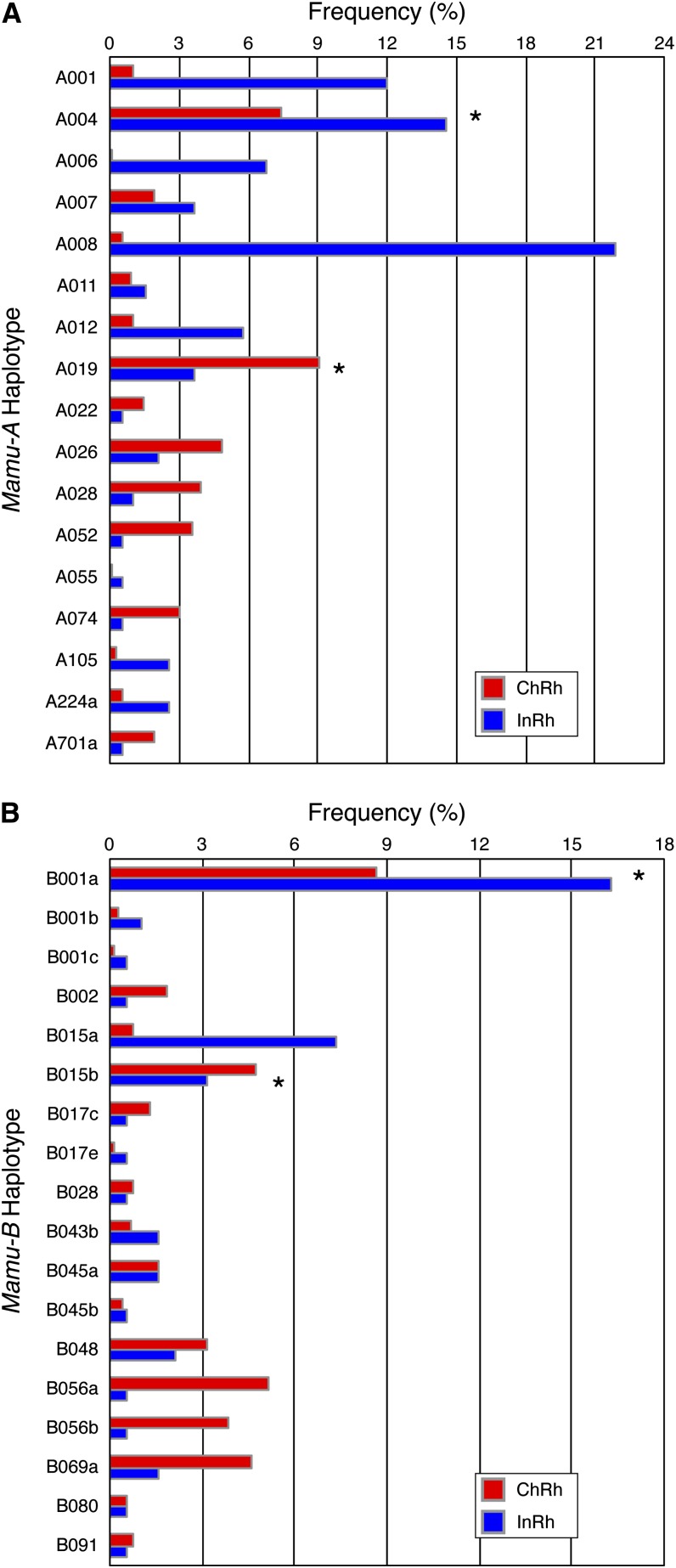
Frequency distribution by population for 35 haplotypes shared between Chinese- and Indian-origin rhesus macaques. (A) Average frequencies by population for the 17 shared *Mamu-A* haplotypes observed in the full cohort of 385 Chinese-origin and 96 Indian-origin rhesus macaques. Frequencies were calculated based on total number of chromosomes examined (770 Chinese and 192 Indian chromosomes) to account for each animal having two haplotypes for each region. Asterisks indicate haplotypes with an average frequency greater than 3% for both populations. (B) Average frequencies by population for the 18 shared *Mamu-B* haplotypes. Asterisks indicate haplotypes with an average frequency greater than 3% for both populations.

**Figure 5:**
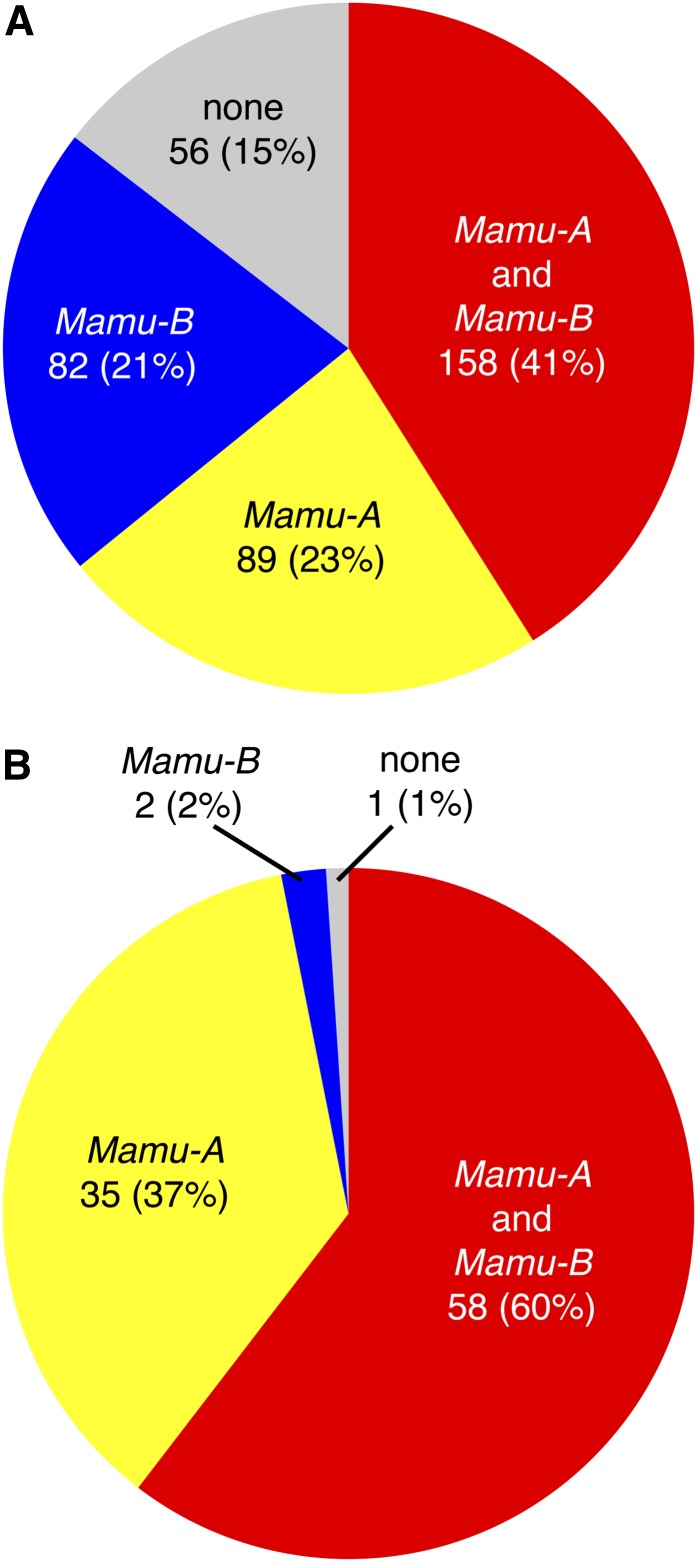
Abundance of shared MHC class I haplotypes in Chinese- and Indian-origin rhesus macaques. (A) Percentage of animals carrying one or more shared MHC class I haplotypes for the full 385 Chinese rhesus macaque cohort, separated by region (expressing both a shared *Mamu-A* and a shared *Mamu-B* haplotype, *Mamu-A* alone, or *Mamu-B* alone). Percentages were calculated based on total number of animals examined. (B) Percentage of animals carrying one or more shared MHC class I haplotypes for the 96 Indian rhesus macaque cohort, separated by region.

The frequencies of these 35 shared haplotypes in each population are shown in Figure 4; frequencies were again based on total number of chromosomes examined. Although several of these shared haplotypes were not among the most abundant in this study, there were four haplotypes (A004, A019, B001a, and B015b) observed at a chromosomal frequency of at least 3% in each population. Remarkably, 85% of the Chinese rhesus macaques in this study express at least one of the 35 shared *Mamu-A* or *Mamu-B* haplotypes (64% carry at least one of the 17 shared *Mamu-A* haplotypes, and 62% carry one or more of the 18 shared *Mamu-B* haplotypes). Likewise, 99% of the Indian rhesus macaques in this cohort express at least one haplotype shared with Chinese-origin animals (Figure 5). Therefore, there is a high likelihood that these shared haplotypes were present in prior studies using Indian-origin rhesus macaques that researchers might reproduce or expand upon today using Chinese-origin rhesus macaques. MHC class I genotyping would need to be performed to confirm presence of shared haplotypes between populations for any specific studies.

### Rhesus macaque haplotypes shared with Mauritian cynomolgus macaques

We also compared our 139 Chinese- and Indian-origin rhesus macaque haplotypes against the well-characterized panel of Mauritian cynomolgus macaque (*Macaca fascicularis*; *Mafa*) haplotypes to explore possible ancestral haplotype sharing between the two species (Wiseman *et al.* 2007; Budde *et al.* 2010). Mauritian cynomolgus macaques exhibit extremely limited MHC class I diversity, as the population arose from a small group of founder cynomolgus macaques deposited on the island in the 1500s (Lawler *et al.* 1995). The MHC class I diversity of Mauritian cynomolgus macaques is effectively described by five *Mafa-A* and seven *Mafa-B* haplotypes (one *Mafa-A* haplotype is commonly associated with three distinct *Mafa-B* haplotypes) (Wiseman *et al.* 2007; Budde *et al.* 2010). Three of our *Mamu-A* haplotypes (A033, A060, and A063) and two of our *Mamu-B* haplotypes (B045a and B150) were homologous to Mauritian cynomolgus macaque haplotypes (Table S1). Four of these haplotypes were observed exclusively in Chinese rhesus macaques; the fifth haplotype (B045a) was detected in both Chinese-origin and Indian-origin rhesus macaques.

## Discussion

It has previously been shown that the specific MHC class I alleles expressed by Chinese- and Indian-origin rhesus macaques are largely distinct, and those distinctions were thought to equate to an unfathomable MHC class I diversity between the two geographically divided populations (Otting *et al.* 2007, 2008; Karl *et al.* 2008; Wu *et al.* 2008; Ma *et al.* 2009; Wiseman *et al.* 2009; Naruse *et al.* 2010; Doxiadis *et al.* 2011). However, our model of defining MHC class I haplotypes of coinherited highly expressed major alleles provides a novel approach for assessing putatively functional similarities between macaque populations. Most notably, more than two thirds of the haplotypes observed in Indian rhesus macaques were shared with Chinese rhesus macaques. This indicates that ancestral haplotypes were retained in both populations after the split from their most recent common ancestor ~162,000 years ago, despite subsequent shrinking and expansion of the Indian and Chinese rhesus macaque populations, respectively (Hernandez *et al.* 2007). Although Chinese rhesus macaques overall display a greater repertoire of MHC class I haplotypes private to the population than Indian rhesus macaques, 85% of the Chinese rhesus macaques in this study express at least one of the shared ancestral *Mamu-A* or *Mamu-B* haplotypes described here (Figure 5). These homologous haplotypes may provide a valuable resource for cross-population studies of functional CD8^+^ T-cell immune response to pathogens.

Does conservation of MHC class I haplotypes correspond to conservation of CD8^+^ T-cell immune responses? Though this remains to be tested at the haplotype level, a previous study demonstrated that it was possible to create Mamu-B*017:03 (a Chinese rhesus macaque-specific allelic variant of the Mamu-B*017 lineage) monomers and tetramers with the SIV Nef_165-173_ peptide IW9 (Ouyang *et al.* 2009). This amino acid sequence was characterized as a CTL epitope restricted by Mamu-B*017:01 in Indian rhesus macaques, an allele that has been associated with control of SIVmac239 viral replication (Mothe *et al.* 2002; Yant *et al.* 2006). Mamu-B*017:03 differs from Mamu-B*017:01 by four amino acids, three of which occur in the peptide binding domain encoded by exons 2-3. These amino acid substitutions did not appear to alter the ability of Mamu-B*017:03 to bind SIV Nef_165-173_ peptide IW9 (Ouyang *et al.* 2009). If the highly similar allelic variants expressed on homologous haplotypes are also able to bind the same peptides, it is very likely those haplotypes would mount similar CD8^+^ T-cell responses in both Chinese- and Indian-origin rhesus macaques. To extend these observations, class I alleles of specific interest sharing a lineage designation would need to be analyzed for predicted binding preferences as well as the functional ability to present specific peptides.

There is also early evidence that sharing of homologous haplotypes may be a pan-macaque phenomenon. Comparison of the rhesus macaque haplotypes identified in this study to an established panel of Mauritian cynomolgus macaque haplotypes revealed a total of five putative orthologous haplotypes, including three of the five *Mafa-A* haplotypes. Interestingly, the cynomolgus macaque haplotypes with putative rhesus macaque orthologs account for more than 62% and 20% of the *Mafa-A* and *Mafa-B* diversity, respectively, in Mauritian cynomolgus macaques (Budde *et al.* 2010). It may therefore be possible to also explore whether rhesus and cynomolgus macaques with conserved ancestral MHC class I haplotypes mount similar CD8^+^ T-cell immune responses. Additional haplotype similarities are also likely to exist between rhesus macaques and cynomolgus macaques of other geographic populations, or between rhesus macaques and other macaque species, but a current lack of defined haplotypes for these other populations make comparisons difficult.

Overall, this study provides the first large-scale description of MHC class I transcript-based haplotypes in rhesus macaques. It begins to explore some of the variability of haplotypes observed between independent experimental research cohorts of Chinese rhesus macaques, and provides a valuable chromosomal haplotype frequency survey for groups looking to study high-frequency Chinese rhesus macaque alleles. Importantly, it highlights the unexpected sharing of Indian rhesus macaque haplotypes with rhesus macaques from China, which may facilitate comparisons of MHC class I−mediated response to pathogens across populations.

## Supplementary Material

Supporting Information
