# Supplementary material for: Major Histocompatibility Complex Class I Haplotype Diversity in Chinese Rhesus Macaques
Source: G3 (Bethesda). 2013 Jul 1;3(7):1195–201. doi: 10.1534/g3.113.006254 (PMC3704247; doi:10.1534/g3.113.006254)
Supplement: Supporting Information [file supp_3_7_1195__index.html]

Major Histocompatibility Complex Class I Haplotype Diversity in Chinese Rhesus Macaques — Supporting Information 

# Major Histocompatibility Complex Class I Haplotype Diversity in Chinese Rhesus Macaques

## Supporting Information for Karl *et al.*, 2013

**Files in this Data Supplement:**

- Supporting Information - Figure S1, File S1, and Tables S1-S3 (PDF, 255 KB)
- Figure S1 - Galaxy-based data analysis workflow (PDF, 212 KB)
- File S1 - Supplemental Materials and Methods (PDF, 110 KB)
- Table S1 - MHC class I *Mamu-A* and *Mamu-B* haplotypes from Chinese- and Indian-origin rhesus macaques (.xls, 86 KB)
- Table S2 - Summary of MHC class I *Mamu-A* and *Mamu-B* haplotypes observed in each macaque (.xls, 101 KB)
- Table S3 - Allelic variants identified in the core set of 51 Chinese-origin and 96 Indian-origin rhesus macaques (.xls, 111 KB)
