# Supplementary material for: Major Histocompatibility Complex Class I Haplotype Diversity in Chinese Rhesus Macaques
Source: G3 (Bethesda). 2013 Jul 1;3(7):1195–201. doi: 10.1534/g3.113.006254 (PMC3704247; doi:10.1534/g3.113.006254)
Supplement: Supporting Information [file supp_g3.113.006254_006254SI.pdf]

G3: Genes | Genomes | Genetics

Major histocompatibility complex class I haplotype diversity in Chinese rhesus macaques

Julie A. Karl[*], Patrick S. Bohn[*], Roger W. Wiseman[*], Francesca A. Nimityongskul[*], Simon M. Lank[*], Gabriel J. Starrett[*], and David H. O'Connor[*,§]

[*] Wisconsin National Primate Research Center, University of Wisconsin-Madison, Madison, WI, 53715
[§] Department of Pathology and Laboratory Medicine, University of Wisconsin-Madison, Madison, WI, 53705

Corresponding Author: David H. O'Connor, Department of Pathology and Laboratory Medicine, University of Wisconsin-Madison, 555 Science Drive, Madison, WI, 53711. Phone: 608-890-0845. Fax: 608-265-8084. E-mail: doconnor@primate.wisc.edu

DOI: **10.1534/g3.113.006254**

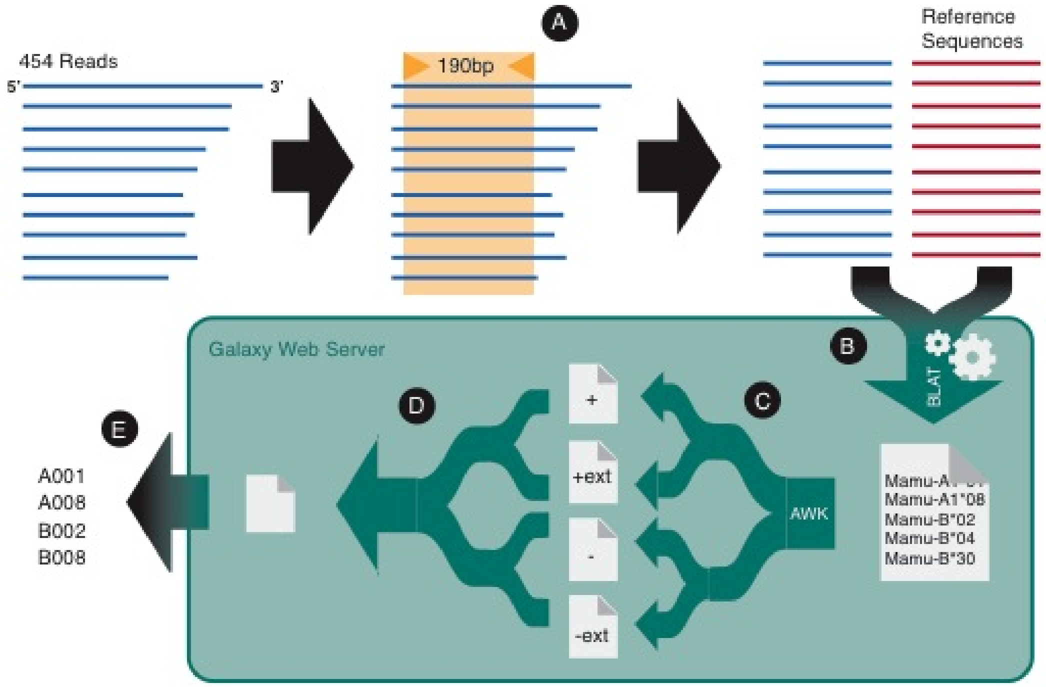

Figure S1 Galaxy-based data analysis workflow. (A) Roche/454 reads were uniformly trimmed down to 190 bp, removing the 5' multiplex identifier (MID) sequence and trimming the remaining length from the 3' end. (B) Reads and reference sequences were uploaded into our Galaxy web server and processed through the BLAST-like analysis tool (BLAT). (C) High-quality, full-length matches were extracted from the BLAT output and binned by direction. (D) Matches were grouped by sample, counted and merged into one line per allele. (E) The report file was then processed manually to generate the haplotype calls.

File S1

Supplemental Materials and Methods

RNA isolation

RNA was isolated from whole blood or peripheral blood mononuclear cell samples using the Roche MagNA Pure LC instrument and RNA High Performance kit (Roche Applied Science, Indianapolis, IN, USA), following manufacturer's protocols. Up to 32 animals were processed simultaneously. RNA samples were quantitated upon completion of the isolation run with a NanoDrop (Thermo Fisher Scientific, Waltham, MA, USA) prior to proceeding with cDNA synthesis. Typical RNA concentrations from robotic isolation ranged between 5-35ng/µl with a 50µl elution volume.

Synthesis of first-strand cDNA

cDNA was synthesized using the Superscript III First-Strand Synthesis System for RT-PCR kit (Invitrogen, Carlsband, CA, USA) essentially following manufacturer's protocols. In brief, a reaction mixture of 1µl 10mM dNTP mix, 1µl 50µM Oligo(dT)$_{20}$, and 50ng total RNA (or up to 8µl if 50ng RNA would exceed 8µl) was prepared for each sample. If necessary, DEPC-treated water was added to obtain a 10µl final reaction volume. Reactions were incubated at 65°C for 5 minutes to denature. During this incubation, the cDNA synthesis master mix was prepared (consisting of 2µl 10X RT Buffer, 4µl 25mM $MgCl_2$, 2µl 0.1M DTT, 1µl RNaseOUT, and 1µl SuperScript III RT per sample). After denaturation, the initial reaction mixture was held at 4°C while 10µl cDNA synthesis master mix was added to each sample. Samples were then incubated at 50°C for 50 minutes, followed by a 5 minute incubation at 85°C to terminate the reaction. Finally, 1µl 2U/µl *E. coli* RNase H was added to each sample, and samples were incubated at 37°C for 20 minutes to remove any remaining RNA.

PCR amplification of MHC class I products

PCR products were generated using primers specific for the MHC class I region. Primers were designed in highly conserved portions of the class I region spanning either exon 2 (the 248bp amplicon) or exons 2-4 (the 638bp amplicon). All primers were designed to amplify both *Mamu-A* and *Mamu-B* sequences, based on alignments of all rhesus macaque classical class I alleles submitted to GenBank. Roche/454 Life Sciences-specific adapters required for emulsion PCR and sequencing, as well as multiplex identifier (MID) tag sequences were appended to the 5' ends of each oligo at the time of primer synthesis. A unique set of MID tagged primers were used for each animal to molecularly barcode all PCR products. Primer sequences are shown below, with the Roche/454 adaptors in bold. There are currently 153 distinct 10bp MID tag sequences designed by Roche/454 which can be inserted into the portion of the primer marked [MID] (sequences for the MID tags are available upon request).

248 bp (exon 2) PCR primers:

SBT190-F 5'-**CGTATCGCCTCCCTCGCGCCATCAG**[MID]GCTACGTGGACGACACG

SBT190-R 5'-**CTATGCGCCTTGCCAGCCCGCTCAG**[MID]GCTACGTGGACGACACG

638 bp (exon 2-4) PCR primers:

SBT568-F 5'-**CGTATCGCCTCCCTCGCGCCATCAG**[MID]GTGGGCTACGTGGACGAC

SBT568-R 5'-**CTATGCGCCTTGCCAGCCCGCTCAG**[MID]TGATCTCCGCAGGGTAGAAG

High-fidelity Phusion polymerase (New England Biolabs, Ipswich, MA, USA) was used for the PCR reactions, since it has proofreading capabilities allowing small variation under the primer to be tolerated so that the few alleles with known mismatches against the primers can potentially still be amplified. A 50µl primer-specific reaction mixture consisting of 25µl 2X Phusion High-Fidelity PCR Master Mix, 15µl nuclease-free water, 2.5µl 1µM forward PCR primer, 2.5µl 1µM reverse PCR primer, and 5µl cDNA was prepared for each sample. The following PCR cycle conditions were performed on an MJ Research Tetrad Thermocycler (Bio-Rad Laboratories, Hercules, CA, USA): initial denaturation at 98°C for 3 minutes, amplification over 23 cycles of 98°C for 5 seconds, 60°C for 1 second, 72°C for 20 seconds, and a final elongation of 72°C for 5 minutes. Aliquots of each reaction were checked for amplification using the Flash Gel DNA cassette system (Lonza, Basel, Switzerland), and an additional 3-6 cycles of PCR were performed if necessary to generate a visible product band. To reduce the amount of PCR-induced error, including chimeric allele recombination, the total number of cycles for each reaction were kept to a minimum.

Amplicon purification, quantification, and pooling

The AMPure XP PCR Purification kit (Agencourt Bioscience Corporation, Beverly, MA, USA) was used to remove short primer dimer products from each PCR reaction. Beads were used according to manufacturer's protocol, using a 1:1 ratio of PCR reaction to magnetics beads. Two complete purifications were performed per reaction. Post purification, the Quant-iT dsDNA HS Assay kit and a Qubit fluorometer (Invitrogen) were used to quantitate all samples, following the manufacturer's protocol. In brief, a master mix was prepared by combining 199µl Quant-iT buffer with 1µl Quant-iT dye reagent for each sample. Standards were prepared by adding 10µl of each provided standard to 190µl master mix, and 2µl of each sample was added to 198µl master mix, all in individual Qubit tubes. Standards were used to calibrate the fluorometer prior to reading sample concentrations. Products were then all normalized to 0.3ng/µl and pools containing uniquely MID-tagged PCR products were created by adding equal volumes of each sample. Variable numbers of products were pooled together for each sequencer run, depending on the sequencing platform.

High-throughput sequencing

Sequencing for all amplicons was performed using Roche/454 second-generation methods following manufacturer's protocols for emulsion PCR and pyrosequencing (Roche Applied Sciences). The core set of 51 Chinese rhesus macaques were pooled together and this pool was run three times on the GS Junior pyrosequencer. An average of 4,884 reads per animal were obtained in total. The 96 Indian rhesus macaques were separated into two pools of 48 animals/pool and sequenced in two runs on the GS Junior; an average of 874 reads per animal were obtained for this cohort. The expanded set of Chinese rhesus macaques were sequenced on a combination of GS Junior and GS FLX platforms in a total of 25 different pools. Each pool contained from 12-48 samples, depending on the platform used for each run. An average of 1,605 reads per animal were obtained for this set of samples. Read lengths averaged ~200bp for the 248bp amplicon sequencing runs and ~500bp for the 638bp amplicon runs.

Data analysis

For the core set of 51 Chinese-origin and 96 Indian-origin rhesus macaques, 638bp amplicon sequences were analyzed using a combination of the programs Geneious Pro (BioLegend, San Diego, CA, USA) and CodonCode Aligner (CodonCode, Dedham, MA, USA). The sff files for each data set were imported into Geneious Pro (version 5.4) and all sequences were trimmed to fixed 430bp lengths from the 3' end. Sequences were then binned by MID tag, so all reads could be tied back to the specific animal from which they were obtained. Uni-directional contigs of sequence reads were assembled for each animal at 100% stringency over 410bp, so all contigs generated in this step were essentially identical over the available sequence. FASTA format sequences of all contigs for each sample were then exported, and downstream analysis was performed in CodonCode Aligner (version 3.7). Bi-directional reads were assembled at 100% identity, and the ~250bp overlap region between the forward and reverse contigs was interrogated thoroughly to ensure that all assemblies were unambiguous. That is, if a single forward contig was 100% identical in the overlap region to two differing reverse contigs, those three contigs would be excluded from downstream analysis. Additionally, sequences were checked for apparent PCR recombination chimerism. Bi-directional assemblies were then interrogated against a curated database of known rhesus macaque MHC class I alleles using the Basic Local Alignment Search Tool (BLAST). Novel alleles supported by four or more sequence reads were submitted to GenBank (KC205288-KC205396), and were assigned putative names based on closest identity to officially named alleles.

Mamu-A and *Mamu-B* haplotypes were inferred by examining patterns of allele sharing across animals. That is, if two or more animals expressed the same 2+ alleles, those alleles were assumed to be traveling together on a chromosome. Often,

two or more haplotype configurations differed only in specific allele content, not in major allele lineages present on the haplotype. To simplify haplotype designations, alleles were downsampled to lineage level and only 'major' MHC class I alleles were considered. Nonhuman primates express as many as 30 different MHC class I alleles and transcribe those alleles at differing levels. They can be roughly divided into 'major' (highly transcribed) and 'minor' (lesser transcribed) alleles. Our criteria for defining a major allele is that it must exceed a 4% transcript abundance and be observed in greater than 90% of the total samples where it would be predicted based on presence of other alleles associated with the haplotype.

Once putative haplotypes were defined, a simpler workflow was used to assign haplotype designations to the additional 334 Chinese rhesus macaque samples from the expanded set. All individual sequence reads obtained for each sample were trimmed to 190bp and directly compared against the curated database (amended to contain the novel alleles identified from the core set of Chinese rhesus samples). That comparison was performed using BLAST-like analysis tool (BLAT) in a local instance of Galaxy (Penn State/Emory, State College/Atlanta, PA/GA, USA). Four haplotypes (two *Mamu-A* and two *Mamu-B*) were assigned for each animal by identifying groups of diagnostic, shared alleles from the previously defined putative haplotypes.

The Venn diagram for Figure 3 was created based on images generated using free online software (http://bioinforx.com/free/bxarrays/venndiagram.php).

Tables S1-S3

Available for download at http://www.g3journal.org/lookup/suppl/doi:10.1534/g3.113.006254/-/DC1

Table S1 MHC class I *Mamu-A* and *Mamu-B* haplotypes from Chinese- and Indian-origin rhesus macaques.

Table S2 Summary of MHC class I *Mamu-A* and *Mamu-B* haplotypes observed in each macaque.

Table S3 Allelic variants identified in the core set of 51 Chinese-origin and 96 Indian-origin rhesus macaques.
