# Supplementary material for: Major Histocompatibility Complex Class I Haplotype Diversity in Chinese Rhesus Macaques
Source: G3 (Bethesda). 2013 Jul 1;3(7):1195–201. doi: 10.1534/g3.113.006254 (PMC3704247; doi:10.1534/g3.113.006254)
Supplement: Supporting Information [file supp_g3.113.006254_FigureS1.pdf]

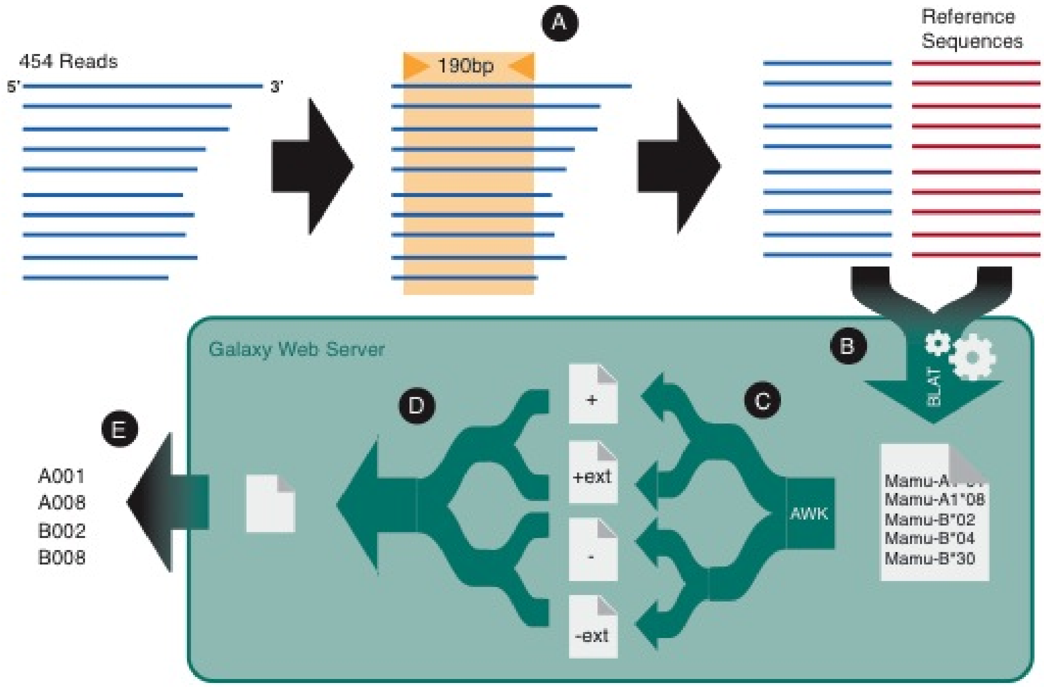

Figure S1 Galaxy-based data analysis workflow. (A) Roche/454 reads were uniformly trimmed down to 190 bp, removing the 5' multiplex identifier (MID) sequence and trimming the remaining length from the 3' end. (B) Reads and reference sequences were uploaded into our Galaxy web server and processed through the BLAST-like analysis tool (BLAT). (C) High-quality, full-length matches were extracted from the BLAT output and binned by direction. (D) Matches were grouped by sample, counted and merged into one line per allele. (E) The report file was then processed manually to generate the haplotype calls.
